# Aptamer Paper-Based Fluorescent Sensor for Determination of SARS-CoV-2 Spike Protein

**DOI:** 10.3390/s25061637

**Published:** 2025-03-07

**Authors:** Jincai Yang, Zunquan Zhao, Tianyi Ma, Jialei Bai

**Affiliations:** Tianjin Key Laboratory of Risk Assessment and Control Technology for Environment and Food Safety, Military Medical Sciences Academy, Academy of Military Sciences, Tianjin 300050, China; jiasang27@163.com (J.Y.); zhaozunq2009@163.com (Z.Z.); matianyi2023@foxmail.com (T.M.)

**Keywords:** SARS-CoV-2, fluorescent detection, paper-based assay, spike protein, aptamer

## Abstract

Point-of-care (POC) antigen detection plays a crucial role in curbing the spread of viruses. Paper-based fluorescence aptasensors are expected to offer a low-cost tool to meet the needs of decentralized POC diagnosis. Herein, we report on a fluorescent paper-based sensing system for detecting the SARS-CoV-2 spike protein. The sensing system was constructed by loading multi-layer Nb_2_C MXene nano-quenchers and carbon-dot-labeled aptamer (G-CDs@Apt) probes onto a mixed cellulose ester (MCE) paper substrate. On the Nb_2_C MXene/G-CDs@Apt sensing paper, abundant G-CDs@Apt probes were attached to the multilayer MXene nano-quenchers and kept in a fluorescence-off state, while recognition of the target detached the G-CDs@Apt probes formed the nano--quenchers, resulting in fluorescence recovery of the sensing paper. The developed paper-based sensor performed well in the one-step detection of the SARS-CoV-2 S1 protein with a detection limit of 0.067 ng/mL (0.335 pg/test). The assay exhibited good selectivity and anti-interference in the detection of the SARS-CoV-2 S1 protein in artificial saliva. Moreover, the paper-based aptasensor was successfully used to detect the SARS-CoV-2 S1 protein in actual environmental samples with recoveries of 90.87–100.55% and relative standard deviations of 1.52–3.41%. The proposed technology provides a cost-effective alternative to traditional antibody test strips for a wide range of POC diagnostic applications.

## 1. Introduction

Since its emergence in late 2019, the severe acute respiratory syndrome coronavirus 2 (SARS-CoV-2)-induced coronavirus disease 2019 (COVID-19) pandemic caused more than 7 million deaths worldwide [1]. Given the growing threat of acute respiratory viral infections to public health, rapid and accurate diagnosis tools, especially point-of-care (POC) testing, play a paramount role in curbing the spread of the virus [2,3]. In general, coronavirus diagnosis strategies mainly include nucleic acid, antibody, and antigen detection [4]. Among these methods, nucleic acid and antigen detection can directly detect the presence of an ongoing infection, targeting viral RNA and proteins, respectively. Reverse transcriptase-polymerase chain reaction (RT-PCR) testing is widely used for the diagnosis of COVID-19 because of its high accuracy and sensitivity, but it relies on expensive instruments and professional operators [3,5]. Moreover, most molecular diagnostic tools involving isothermal nucleic acid amplification require a laboratory operating environment to minimize the aerosol contamination of the amplification products [6]. In contrast, antigen assays, which do not amplify the target, are the most common methods of accessible and decentralized molecular testing [1,2].

SARS-CoV-2 is mainly composed of four structural proteins: spike (S), nucleocapsid (N), membrane (M), and envelope (E) proteins [7]. Among them, the S protein is displayed as a trimeric membrane glycoprotein located on the surface of the virion, consisting of an S1 attachment subunit and S2 fusion subunit [8]. The receptor binding domains (RBD) at the S1 subunit can bind to human angiotensin-converting enzyme 2 (hACE2) with high affinity, facilitating viral entry into host cells [9]. With high immunogenicity and specificity, the SARS-CoV-2 spike protein or its S1 subunit has become the most suitable antigen for the direct detection of live and infectious virus particles [10]. Furthermore, the SARS-CoV-2 spike protein is considered to be a key biomarker for the so-called “long COVID” syndrome or post-acute sequelae of COVID-19 (PASC), which contributes to fibrin/amyloid microclot formation or vasculature damage [11]. A retrospective pilot study found that spike proteins were detected in 60% of PASC participants within 12 months of COVID-19 onset, whereas no spikes were detected in COVID-19 convalescent control patients [12]. Therefore, much effort has been put into developing spike antigen detection methods in recent years. A fiber-optic biolayer interferometer-based biosensor was developed for the automated detection of SARS-CoV-2 in 13 min using a specific anti-S1 antibody pair [13]. Another team employed immuno-sensors based on organic electrochemical transistors (OECTs) to monitor spike proteins as the biomarker of long COVID syndrome [14]. Liu et al. introduced a phage-expressed peptide specific to the SARS-CoV-2 spike protein into the construction of a “peptide–antigen–antibody” ELISA assay [3]. Through tyramine signal amplification, this method achieved ultrasensitive detection of femtomolar level SARS-CoV-2 antigen. Nevertheless, the limitations of antibodies or peptides as recognition elements should not be ignored, such as their high cost, short lifespan, and difficulty in surface modification.

Paper-based analytical devices offer excellent prospects for use in POC diagnostics as inexpensive, readily available, and user-friendly testing tools [15]. Particularly in resource-limited settings where laboratory infrastructure, skilled personnel, and financial support are lacking, paper-based diagnostics have immense potential for initiating and scaling up on-site medical services for the prevention and control of infectious diseases. Most paper-based sensors use costly antibodies as recognition elements, hindering their application in at-home testing. Aptamer-based detection papers have the potential to offer an affordable and cost-effective solution to meet decentralized POC diagnostic demands. Nucleic acid aptamers have been widely used as candidates to recognize or capture targets in a variety of biosensing applications [16,17]. The advantages of these screened oligonucleotide chains over antibodies include lower chemical synthesis costs, better physicochemical stability, and simpler modification steps, making them more suitable for frequent testing in at-home scenarios [18]. Thus, several strategies have been successfully developed for the construction of aptasensors towards spike antigen, such as electrochemical biochips [18], electrochemiluminescence platforms [19], and surface enhanced Raman scattering (SERS)-based biosensors [5]. In these designs, aptamers or hybridized duplexes containing aptamers are often firmly immobilized on the surface of the electrode or substrate, efficiently performing functions such as signal recognition, signal transmission, and signal amplification [5,18,19,20]. Due to the potential effects of the immobilized state on aptamer conformation, the recognition capacity of the aptamer may be affected [21]. In comparison, aptamers in the free state can bind analytes more efficiently with advantages such as small size, low steric hindrance, and good dispersibility. Recently, a nano-quenching and recovery detector for the CD44 antigen was developed by utilizing multi-layer black phosphorus nanosheets (BPNSs) and a dispersed Cy3-labelled CD44 aptamer [22]. The aptamers can attach to BPNSs via the van der Waals force and detach from BPNSs due to the target recognition reaction. The detector-enabled visual detection of CD44 expression on cancer cells for cancer drug resistance monitoring works in this manner.

**Figure 1:**
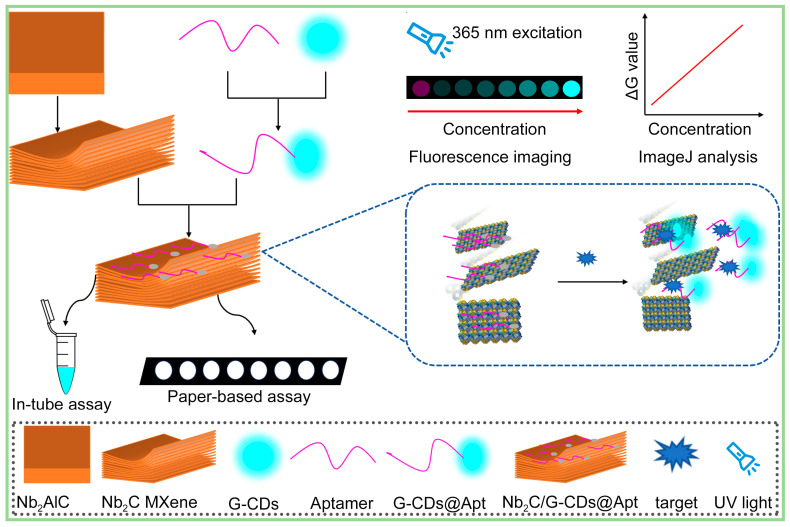
Schematic of aptamer-based fluorescence paper sensor for SARS-CoV-2 spike protein.

In this work, we aimed to develop an aptamer-based fluorescence paper sensor for low-cost, sensitive, and one-step detection of the SARS-CoV-2 spike protein. Our proposed paper-based sensor was obtained by loading a mixed cellulose ester (MCE) membrane substrate with a fluorescence quenching recovery system consisting of MXene nano-quenchers and carbon quantum dot-labeled aptamers. Figure 1 shows the sensing strategy and the fundamentals of fluorescence imaging. Niobium carbide (Nb_2_C) MXene nano-quenchers with wafer cookie-like multilayered nanostructures were made from niobium–aluminum carbide (Nb_2_AlC) via hydrofluoric acid solution etching. Multilayered Nb_2_C MXene nano-quenchers adsorbed a wealth of aptamer probes and could be loaded on MCE paper for on-site detection. The aptamers were functionalized with green fluorescence carbon quantum dots (G-CDs), maintaining the advantages of the small size and low steric hindrance of the fluorescent aptamer probes. These aptamer probes (G-CDs@Apt) adsorbed on MXene nano-quenchers were in a fluorescence-quenched state due to the fluorescence resonance energy transfer effect between MXene and the G-CDs. When the spike protein was present, the aptamer probes moved away from the MXene because it bound more readily to the spike protein. Subsequently, the unattached probes were dispersed in the MXene-free region of the paper substrate, leading to fluorescence recovery. Through fluorescence imaging under a UV light, visual fluorescence detection of spike protein can be achieved. The G channel values of paper-based fluorescence imaging can be quantified by ImageJ software. In addition, the background noise in the G (green) channel was very low compared with the green fluorescence signals of the released G-CDs@Apt probes.

## 2. Materials and Methods

### 2.1. Reagents and Apparatus

Recombinant SARS-CoV-2 S1 protein, bovine serum albumin (BSA), hemoglobin (HB), and SARS-CoV-2 nucleocapsid protein were obtained from Sangon Biotech Co., Ltd. (Shanghai, China). Green carbon quantum dots were supplied by Mesolight Co., Ltd. (Suzhou, China). 1-(3-Dimethylaminopropyl)-3-ethylcarbodiimide hydrochloride (EDC), N-Hydroxysuccinimide (NHS), sterilized phosphate buffer solution (PBS, 100 mM, pH = 7.4), and hydrofluoric acid (HF) were purchased from Aladdin Reagent Co., Ltd. (Shanghai, China). SARS-CoV-2 S-RBD aptamer was supplied by Sangon Biotech Co., Ltd. (Shanghai, China), and the sequence was as follows [23]: 5′-NH_2_-C_6_-CAGCACCGACCTTGTGCTTTGGGAGTGCTGGTCCAAGGGCGTTAATGGACA-3′. Niobium carbide aluminum (Nb_2_AlC) powder was purchased from McLean Reagent Co., Ltd. (Shanghai, China). Ultrapure water was supplied from a Millipore water purification system (≥18 MΩ, Milli-Q, Millipore, MA, USA). Mixed cellulose ester (MCE) paper was obtained from Jinteng Experiment Equipment Co., Ltd. (Tianjin, China). Artificial saliva was purchased from Yuanye Bio-Technology Co., Ltd. (Shanghai, China). All other chemicals were analytical grade and can be used without further purification. More detailed information is provided in Table S1. Apparatus used in the study included KQ-500DE ultrasonic cleaner (Kunshan Ultrasonic Instrument Co., Ltd., Suzhou, China), F97pro fluorescence spectrophotometer (Shanghai Lengguang Technology Co., Ltd., Shanghai, China), Tu-1901 ultraviolet–visible (UV–vis) absorption spectrophotometer (Beijing Purkinje GENERAL Instrument Co., Ltd., Beijing, China), and Regulus 8100 scanning electron microscope (SEM) (Hitachi High-Tech Instruments Co., Ltd., Tokyo, Japan).

### 2.2. Synthesis of Nb_2_C MXene Nano-Quenchers

Nb_2_C MXene nano-quenchers were synthesized by removing the aluminum layer of Nb_2_AlC by HF etching [24,25]. Briefly, Nb_2_AlC powder (1 g) was slowly added in batches to 40 mL of HF (40%) solution over a 10 min period, then stirred in a 60 °C-water bath for 24 h. The precipitate was collected by centrifugation at 10,000 rpm for 10 min. The precipitate was then mixed with 10 mL of HF (40%) and sonicated for 13 h. After centrifugation at 10,000 rpm for 10 min, the precipitate was collected and washed to neutrality with ultrapure water. Finally, the precipitate was frozen at −80 °C for 12 h and then lyophilized to obtain Nb_2_C MXene powder, which can be stored for an extended period at 4 °C under nitrogen protection.

### 2.3. Preparation of Fluorescent Aptamer Probes

First, 5 mg of EDC and 5 mg of NHS were dispersed in 1 mL of PBS buffer (100 mM, pH = 7.4). Then, 100 μL of G-CDs (0.5–4 mg/mL) was mixed with 100 μL of the above EDC/NHS solution and sonicated at room temperature for 30 min. Next, 20 μL of the post-sonication solution was mixed with 80 μL of the amino-modified aptamer (5 μM) and incubated at 37 °C for 12 h. The solution was then filtered (10,000 rpm, 5 min) in an ultrafiltration tube (10 kDa) to obtain the fluorescent aptamer probes (G-CDs@Apt).

### 2.4. Detection Method in Tube

A total of 50 μL of ultrapure water, 10 μL of PBS buffer (100 mM, pH = 7.4), 10 μL of G-CDs@Apt solution, and 10 μL of Nb_2_C-MXene solution (1.5 mg/mL) were added to the reaction tube, mixed well, and then incubated for 25 min at 37 °C in a horizontal shaker away from light. After adding 20 μL of different concentrations of the SARS-CoV-2 S1 protein samples, the reaction tubes were incubated in the dark at 37 °C for 25 min and the fluorescence intensities were measured under 470 nm excitation by an F97pro fluorescence spectrophotometer.

### 2.5. Paper-Based Detection System

A facile paper-based analytical device was constructed consisting of a UV-lamped dark box, 8-well enzyme-linked strips, and aptasensor-loaded MCE test papers. The test paper specifically recognized the target and emitted green fluorescence under 365 nm UV light, enabling qualitative and quantitative detection of SARS-CoV-2 S1 protein. Specifically, 4 mm diameter paper disks were obtained by utilizing a 4 mm biopunch to punch holes in the MCE paper and placed on the bottom side of the enzyme-linked strips. Then, 50 μL of ultrapure water, 10 μL of PBS buffer (100 mM, pH = 7.4), 10 μL of G-CDs@Apt solution, and 10 μL of Nb_2_C solution (1.5 mg/mL) were added to a reaction tube, mixed well, and incubated for 25 min in the dark. A total of 5 μL of the above incubation solution (Nb_2_C/G-CDs@Apt) was dropped onto each MCE disc and dried in the dark. When used for detection, 5 μL of samples or the vehicle were added dropwise to the discs and incubated for 25 min, followed by detection under 365 nm UV excitation in the dark box. The fluorescence images of the test papers were recorded by a mobile phone, and the G channel values were obtained using ImageJ software (Version 1.54d).

### 2.6. Real Sample Analysis

Surface water from Haihe River (Tianjin, China) was selected as urban water environmental samples to investigate the practicability of the developed test paper. Prior to analysis, water samples were filtered through a 0.22 μm filter and subsequently centrifuged at 4000 rpm for 5 min to remove precipitates [26].

## 3. Results and Discussion

### 3.1. Multi-Layer Nb_2_C MXene Nano-Quenchers Loaded on MCE Paper Substrate

**Figure 2:**
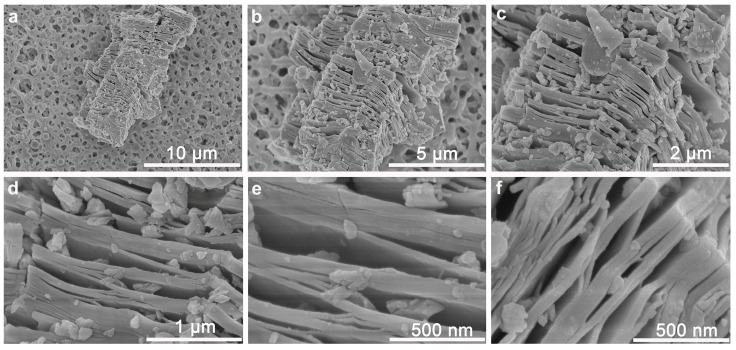
(**a**–**f**) SEM images of Nb_2_C MXene nano-quenchers on MCE substrate. Scale size: (**a**) 10 μm, (**b**) 5 μm, (**c**) 2 μm, (**d**) 1 μm, (**e**) 500 nm, and (**f**) 500 nm.

The strategy we employed was to utilize a nano-quencher to adsorb the aptamer probe and quench its fluorescence. Common nano-quenchers are mainly thin nanosheets of materials such as graphene oxide [27], molybdenum disulfide [28], and metal-organic frameworks [29], etc., which mostly rely on electrostatic and π–π interactions between the monolayer materials and nucleic acids to achieve adsorption. Due to their thin-layer lamellar nanostructures, they show good detection ability in liquid detection. However, when attaching high-performance nanosheets on solid-phase surfaces, problems such as low probe carrying capacity and easy bending are revealed, requiring these methods to be coupled with other signal amplification means [15]. Here, we used a multi-layer Nb_2_C MXene material as the nano-quencher to be loaded on the paper substrate (Figure 1). The Nb_2_C MXene nano-quencher adsorbed a wealth of fluorescent aptamer probes and quenched the fluorescence. When the target was present, the aptamer recognition of the target induced the aptamer probes to escape from the Nb_2_C MXene nano-quencher, leading to fluorescence recovery. Compared to the bottom-up synthesis approaches which often yield some few-layer nanomaterials, multilayer MXene can be obtained via acid etching methods and serves as a typical two-dimensional (2D) nanomaterial with multilayered nanostructures and abundant interlayer gaps [30]. As shown in Figure 2, multi-layer Nb_2_C MXene nanoblocks were attached to the surface of the MCE paper substrate. After hydrofluoric acid (HF) etching and ultrasonication treatment, the Nb_2_C MXene exhibited a loose stacking structure of multilayered nanosheets with a large number of interlayer gaps (large ones close to 100 nm) of varying sizes, and some material fragments adsorbed on the surface of the material (Figure 2c–f). The Nb_2_C bricks were not uniform in size (1–15 μm), which may be caused by the addition of the ultrasonic treatment during the etching process. Nevertheless, this treatment promoted an increase in the interlayer spacing and an increase in the specific surface area of MXene [24]. During the etching process, the F ions in HF broke the weak Nb-Al bonds in the Nb_2_AlC precursor, leading to the exfoliation of the Al layer, which resulted in the evolution of the MAX phase of the dense Nb_2_AlC nanoblocks into a wafer cookie-like multilayer structure consisting of loosely stacked thick nanosheets (Figures S1 and S2 and Table S2). This nanostructure greatly increases the specific surface area of the material, and the abundant interlayer voids facilitate the effective adsorption and desorption of biomolecules and fluorescent probes. In the case where these nano-quenchers did not overlay the entire paper substrate, fluorescent probes induced by the target to detach from the nano-quencher could land more efficiently onto the quencher-free paper area for fluorescence recovery.

### 3.2. Fluorescence Quenching Due to Absorption of Aptamer Probes by Nb_2_C MXene Nano-Quenchers

**Figure 3:**
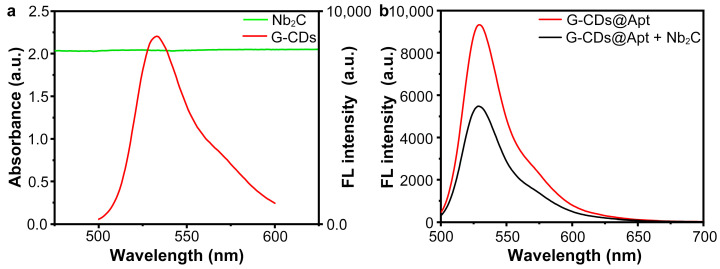
Fluorescence quenching of G-CDs@Apt probes by Nb_2_C MXene nano-quenchers. (**a**) Fluorescence spectra of G-CDs (red line) and UV-vis absorption spectra of Nb_2_C MXene (green line, 0.1 mg/mL). (**b**) Fluorescence spectra of G-CDs@Apt probes in absence or presence of Nb_2_C MXene nano-quenchers under 470 nm excitation.

MXenes are promising candidates for fluorescence-based biosensors due to their excellent properties such as high specific surface area, excellent electrical conductivity, and biocompatibility [31]. Moreover, the terminal groups on the surface of MXene help them to interact with nucleic acid molecules through non-covalent interactions (e.g., hydrogen bonding, ligand bonding, van der Waals forces, and electrostatic interactions) [32]. Thus, the attachment between MXene and fluorophore enables fluorescence quenching by photoinduced electron transfer (PET) [33], Förster resonance energy transfer (FRET) [34], etc. Hong et al. developed a “turn-on” FRET-based detection method for *Vibrio parahaemolyticus* using Ti_3_C_2_ MXene as a quencher and aptamer-modified polyhedral oligomeric silsesquioxane-perovskite quantum dots (POSS-PQDs@Apt) as a probe [34]. In this study, zero-dimensional (0D) green fluorescence carbon dots (G-CDs) were utilized as environmentally friendly water-soluble long-lived fluorophores. As shown in Figure 3a, the fluorescence emission spectra of G-CDs showed a maximum emission peak at 535 nm, while the Nb_2_C MXene (0.1 mg/mL) exhibited a large ultraviolet–visible (UV-vis) absorption capability ranging from 200 nm–800 nm, which overlaps the fluorescence emission of the G-CDs. The result indicated that the Nb_2_C MXene was an ideal candidate for a fluorescence nano-quencher for G-CDs. Further, modification of G-CDs with the SARS-CoV-2 S-RBD aptamer yielded G-CDs@Apt as a recognition probe for the SARS-CoV-2 spike protein. When the G-CDs@Apt probes were mixed with Nb_2_C MXene nano-quenchers, the fluorescence of the G-CDs@Apt probes was significantly diminished, which indicated that the G-CDs@Apts adsorbed on Nb_2_C MXene and induced the fluorescence quenching phenomenon (Figure 3b).

### 3.3. Target-Induced Aptamer Probe Detachment from Nano-Quencher and Fluorescence Recovery

**Figure 4:**
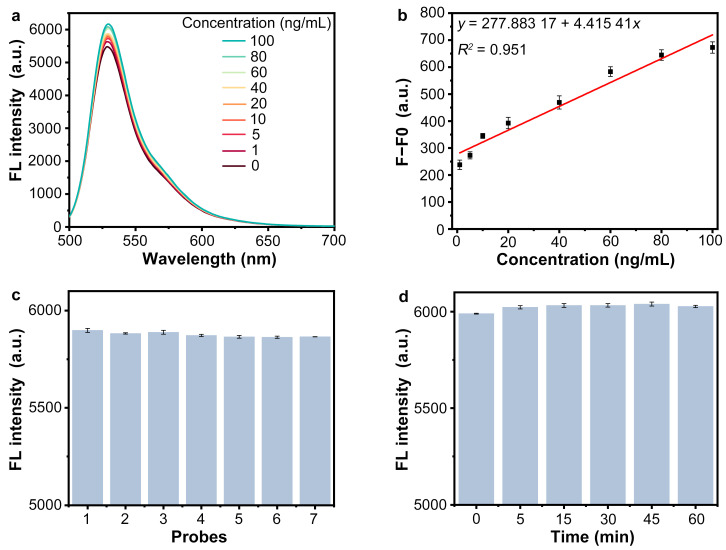
Determination of SARS-CoV-2 S1 protein in a liquid reaction using Nb_2_C MXene/G-CDs@Apt system. (**a**) Fluorescence spectra of Nb_2_C MXene/G-CDs@Apt system after addition of different concentrations of SARS-CoV-2 S1 protein. (**b**) Linear relationship between fluorescence signal change (F−F_0_) and concentration of SARS-CoV-2 S1 protein. (**c**) Fluorescence intensity of different batches of Nb_2_C MXene/G-CDs@Apt system in response to SARS-CoV-2 S1 protein (50 ng/mL). (**d**) Fluorescence intensity of Nb_2_C MXene/G-CDs@Apt system in response to SARS-CoV-2 S1 protein (50 ng/mL) with incandescent pre-exposure for 0–60 min. Excitation: at 470 nm.

In fluorescence quenching recovery aptasensors, the recognition and binding of the aptamer probe to the analyte results in a conformational change of the probe, which allows the probe to escape from the quencher [24,25,35]. Thus, we next tested the response of the Nb_2_C MXene/G-CDs@Apt system to the SARS-CoV-2 spike protein. After adding different concentration gradients of the SARS-CoV-2 S1 protein to the Nb_2_C MXene/G-CDs@Apt system, the fluorescence intensities were measured after 25 min of incubation. As shown in Figure 4a, the fluorescence intensity of the system was enhanced with an increasing SARS-CoV-2 S1 protein concentration. Figure 4b shows a positive linear correlation between the values of fluorescence intensity change before and after the addition of the S1 protein (F−F_0_) and protein concentration ranging from 1 to 100 ng/mL, which can be represented by the equation y = 277.88317 + 4.41541x, R^2^ = 0.951. The detection limit (LOD) was calculated as 0.97 ng/mL according to the 3σ rule. The results suggested that the Nb_2_C MXene/G-CDs@Apt system had the potential to be a “turn-on” fluorescence biosensor for SARS-CoV-2 S1 protein detection. Compared with “turn-off” analytical methods, the turn-on mode can have a higher signal-to-noise ratio and anti-interference capability due to the lower initial background signal [36,37]. To examine the stability of the detection method, we tested the response of different batches of the Nb_2_C MXene/G-CDs@Apt system to a SARS-CoV-2 S1 protein sample at a concentration of 50 ng/mL. As shown in Figure 4c, the fluorescence intensity of the sensing systems prepared from different batches was highly consistent after recognizing the same concentration of analyte, indicating the good stability of the sensing system. On the other hand, we exposed the G-CDs@Apt probe to incandescent light and subsequently tested the Nb_2_C MXene/G-CDs@Apt system in response to the SARS-CoV-2 S1 protein. As shown in Figure 4d, exposing the probe to incandescent light for 0 to 60 min did not significantly affect the sensing performance in detecting the S1 protein, as the fluorescence intensity of the system was not altered by short-term incandescent light exposure. These results indicated that the Nb_2_C MXene/G-CDs@Apt system had good photostability and resistance to photobleaching and was expected to be further applied to paper-based detection methods.

### 3.4. Feasibility of Using Nb_2_C/G-CDs@Apt Test Paper for Detecting SARS-CoV-2 Spike Protein

**Figure 5:**
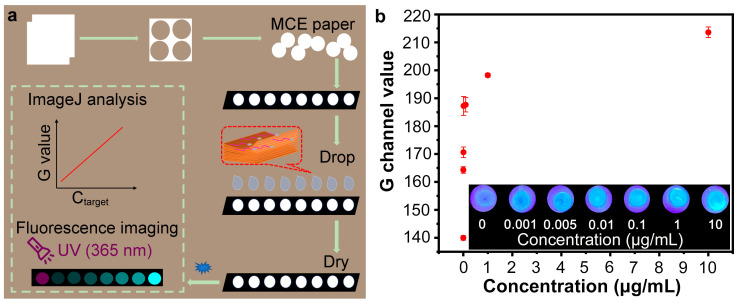
Feasibility of Nb_2_C MXene/G-CDs@Apt sensor for fluorescence paper-based detection of SARS-CoV-2 S1 protein detection. (**a**) Schematic of fabrication and utilization process of paper fluorescence sensor. (**b**) G channel values in reaction region of Nb_2_C MXene/G-CDs@Apt paper sensor after reaction with different concentrations of SARS-CoV-2 S1 protein were analyzed using ImageJ software. Inset: Fluorescence images of Nb_2_C MXene/G-CDs@Apt test paper after reaction with different concentrations of SARS-CoV-2 S1 protein for 25 min under UV light (365 nm).

To verify the feasibility of paper-based detection using the Nb_2_C MXene/G-CDs@Apt system, we prepared the sensing system and loaded it onto the MCE paper substrate as a ready-to-use paper sensor. Different concentrations of the SARS-CoV-2 S1 protein were added to the paper sensor, and after 25 min of reaction, the fluorescence image of the paper sensor under UV light was recorded with a mobile phone. As shown in Figure 5b, the green fluorescence of the paper sensor gradually recovered as the concentration of the SARS-CoV-2 S1 protein increased within a certain range from 0 to 10 μg/mL. This phenomenon suggested that Nb_2_C MXene loaded on the paper substrate adsorbed abundant G-CDs@Apt probes and was in a fluorescence off state, whereas the target induced the release of fluorescent G-CDs@Apt probes to the non-quenching region of the paper substrate, resulting in fluorescence recovery (Figure 5a). Furthermore, the fluorescence intensity of the detection region in the images was quantified by the ImageJ software analysis [38,39], indicating a positive correlation between the G channel value and the concentration of the SARS-CoV-2 S1 protein. Notably, when the SARS-CoV-2 S1 protein was added at a low concentration of 1 ng/mL, there was a significant fluorescence recovery compared to the blank control. These results greatly support the feasibility of the plan to construct a paper-based analysis device by utilizing the Nb_2_C MXene/G-CDs@Apt system for visual detection of the SARS-CoV-2 spike protein. In addition, the fluorescence of the G-CDs and G-CDs@Apt can be excited by blue light (470 nm) as shown in Figure 3 and Figure 4. Thus, CD-based fluorescent probes have the potential to be applied in in vitro or in vivo experiments to identify tumors or biomarkers. However, the in vivo delivery and fluorescence depth sensing of MXene/G-CDs@Apt need more exploration [40].

### 3.5. Optimization of Paper-Based Analytical Method

In order to obtain better performance of the paper-based sensing system, we optimized several experimental conditions including the concentration of the carbon quantum dot, the concentration of Nb_2_C MXene, the incubation time of the Nb_2_C MXene/G-CDs@Apt mixture, and the reaction time after the addition of the SARS-CoV-2 S1 protein.

**Figure 6:**
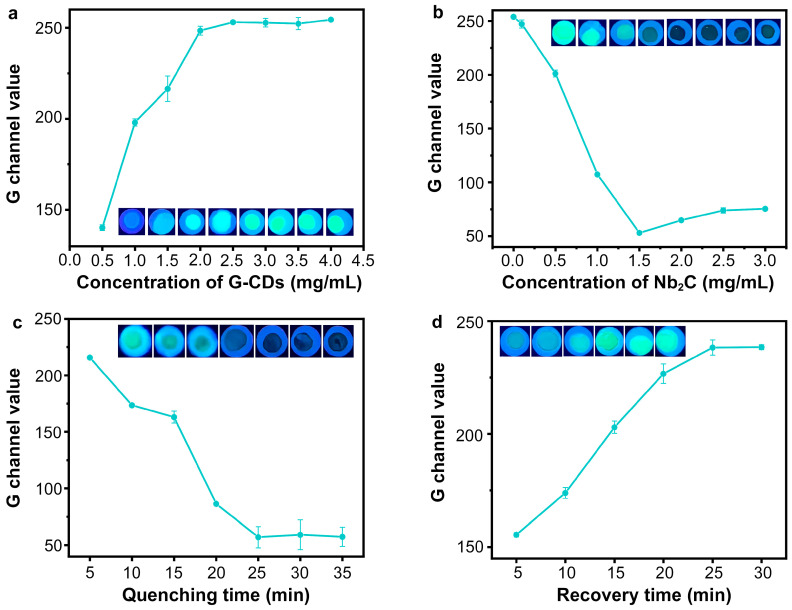
Optimization of experimental conditions for fluorescence paper-based assay. (**a**) Optimization of G-CDs concentration used in aptamer modification process. (**b**) Optimization of Nb_2_C MXene concentration loaded on paper sensor. (**c**) Optimization of pre-incubation time of Nb_2_C MXene/G-CDs@Apt system. (**d**) Optimization of reaction time for fluorescence recovery after addition of SARS-CoV-2 S1 protein.

In this study, carboxyl G-CDs were activated by EDC/NHS and reacted with the amino-modified aptamer to obtain fluorescent aptamer probes. Therefore, the SARS-CoV-2 S-RBD aptamer (5 μM) was modified with different concentrations of carbon dots and loaded onto an MCE paper substrate to examine the effect of modification concentration on the fluorescence intensity of the G-CDs@Apt probe on MCE paper. As shown in Figure 6a, the G channel value of the G-CDs@Apt-probe-loaded MCE paper was enhanced with the increase in the concentration of G-CDs in the modification process. When the concentration of G-CDs reached 2.5 mg/mL, further increasing the modification concentration of G-CDs did not significantly increase the fluorescence intensity of the G-CDs@Apt-probe-loaded MCE paper. Thus, the modification concentration of G-CDs used in subsequent experiments was 2.5 mg/mL. Since the Nb_2_C MXene nano-quencher plays a crucial role in the construction of a paper-based sensing system, we next determined the optimal concentration of Nb_2_C MXene for paper-based analytical detection. As shown in Figure 6b, the G channel value of the paper sensor gradually decreased with the increase in the Nb_2_C MXene concentration, and the quenching effect was the best at 1.5 mg*/*mL of Nb_2_C MXene. Adequate incubation time and reaction time are necessary for excellent sensing performance. Figure 6c showed that the pre-incubation of the mixture of Nb_2_C MXene nano-quencher and G-CDs@Apt probe for 25 min was sufficient to obtain favorable fluorescence quenching. To investigate the effect of reaction time with the addition of analytes, the optimal recovery of fluorescence was observed when the assay reaction time reached 25 min (Figure 6d).

### 3.6. Detection of SARS-CoV-2 Spike Protein by Nb_2_C MXene/G-CDs@Apt Paper Sensor

**Figure 7:**
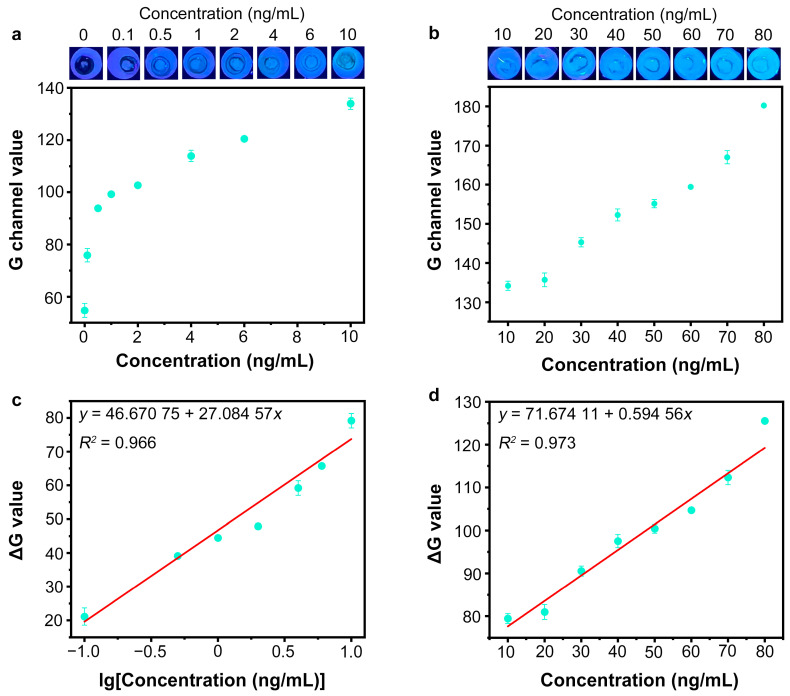
Detection performance of Nb_2_C MXene/G-CDs@Apt fluorescence paper sensor. (**a**) Determination of SARS-CoV-2 S1 protein ranging from 0 to 10 ng/mL using Nb_2_C MXene/G-CDs@Apt test papers. (**b**) Determination of SARS-CoV-2 S1 protein ranging from 10 to 80 ng/mL using Nb_2_C MXene/G-CDs@Apt test papers. (**c**) Linear relationship between ΔG value and logarithm of SARS-CoV-2 S1 protein concentration (0.1–10 ng/mL). (**d**) Linear relationship between ΔG value and SARS-CoV-2 S1 protein concentration (10–80 ng/mL).

The Nb_2_C MXene/G-CDs@Apt paper sensor for SARS-CoV-2 S1 protein determination was used under optimized conditions. A series of different concentrations of the SARS-CoV-2 S1 protein were added to the paper sensor, and the fluorescence images of the detection region after 25 min of reaction are shown in Figure 7a–d. With an increasing amount of the target SARS-CoV-2 S1 protein added, the green fluorescence intensity of detection region was enhanced, resulting from an increase in the released target–aptamer complexes. The green fluorescence of the system had low background noise, and the G-channel values of noise were all below 60 a.u. when analyzed with ImageJ software. Thus, the G channel value of the negative group could be distinguished from that of the positive groups with a target concentration ranging from 0.1 to 80 ng/mL (Figure 7a,b). To further eliminate the effect of green background noise, ΔG (G-G_0_) values were calculated to represent fluorescence recovery levels. As shown in Figure 7c, a linear relationship (y = 46.67075 + 27.08457x, R^2^ = 0.966) was observed between the ΔG value and the logarithm of protein concentration within the range from 0.1 ng/mL to 10 ng/mL. A good linear relationship between the ΔG value and the concentration of the SARS-CoV-2 S1 protein (10 ng/mL to 80 ng/mL) is shown in Figure 7d. The linear equation was expressed as y = 71.67411 + 0.59465x (R^2^ = 0.973). The detection limit was calculated as 0.067 ng/mL (0.335 pg/test) according to the 3σ rule. The specificity of the proposed Nb_2_C MXene/G-CDs@Apt test paper was further investigated by measuring the signal responses towards other proteins including the SARS-CoV-2 N protein, bovine serum albumin (BSA), hemoglobin (HB), and their mixture with the SARS-CoV-2 S1 protein (MIX). The concentration of the SARS-CoV-2 S1 protein was 50 ng/mL, and the concentration of the other proteins was 1 µg/mL. In addition, the proposed Nb_2_C MXene/G-CDs@Apt paper-based sensor was more cost-effective compared to other established analytical methods [41,42,43,44,45,46] (Tables S3 and S4).

**Figure 8:**
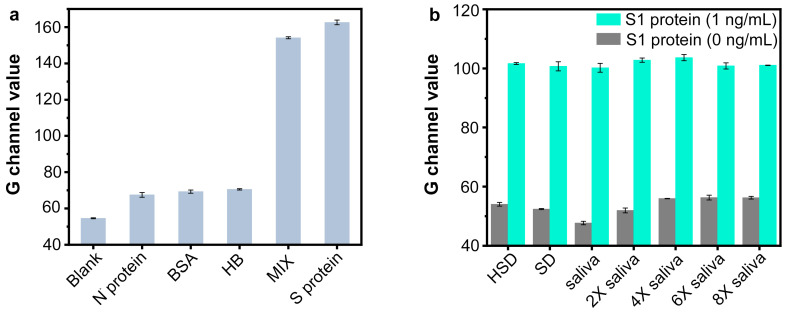
Selectivity and anti-interference of Nb_2_C MXene/G-CDs@Apt fluorescence paper sensor. (**a**) Selectivity of paper-based sensor for SARS-CoV-2 S1 protein towards other proteins. (**b**) Effect of dilution of artificial saliva on SARS-CoV-2 S1 protein detection by sensor. HSD: high-salt phosphate buffer solution (PBS, 100 mM, pH = 7.4) with extra NaCl (274 mM). SD: phosphate buffer solution (PBS, 100 mM, pH = 7.4). “X”: dilution of saliva with ultrapure water.

As shown in Figure 8a, only the SARS-CoV-2 S1 protein and the MIX group resulted in G-channel values above 80 a.u. compared to the other proteins, which indicated that the proposed test paper had excellent selectivity for the SARS-CoV-2 S1 protein. We next applied the paper-based aptasensor to the detection in artificial saliva, to evaluate whether the components in saliva or a sample diluent (SD) buffer affect the sensor performance. Here, the SARS-CoV-2 S1 protein samples were diluted using sterilized phosphate buffer solution (PBS, 100 mM, pH = 7.4) as the SD buffer, using high-salt phosphate buffer solution (PBS, 100 mM, pH = 7.4) with extra NaCl (274 mM) as the HSD buffer, or using artificial saliva with ultrapure water. As shown in Figure 8b, the aptasensor was able to clearly recognize the presence or absence of 1 ng/mL of the S1 protein in the untreated artificial saliva samples. The proposed method also exhibited good anti-interference for high-salt and low-salt detection conditions. In addition, a storage test showed that the Nb_2_C MXene/G-CDs@Apt sensor exhibited good storage stability over a two-week period (Figure S3).

### 3.7. Application in Actual Samples

We further evaluated the use of the Nb_2_C MXene/G-CDs@Apt paper sensor for the detection of the SARS-CoV-2 spike protein in environmental samples. The utility of rivers and wastewater for SARS-CoV-2 surveillance is a promising alternative to community-based testing for COVID-19 [47,48,49]. Researchers collected water samples from up to 112 river or defunct wastewater treatment plant sites in Malawi, and SARS-CoV-2 was detected in 8.3% of the samples [47]. Here, river water samples were collected at Haihe River in Tianjin city as environmental matrices. Recovery tests were performed by adding different concentrations of the SARS-CoV-2 S1 protein (0, 10, 50, and 80 ng/mL) to river water samples and analyzing them with the Nb_2_C MXene/G-CDs@Apt paper sensor. As shown in Table 1, the recovery rates and relative standard deviations (RSD) were 95.96–100.55% and 1.52–3.41%, respectively. The result validated that the constructed method had good practicability for SARS-CoV-2 spike protein detection in actual environmental samples.

## 4. Conclusions

In summary, we established a facile, cost-effective, and sensitive fluorescence “turn−on” paper-based sensor for point-of-care detection of the SARS-CoV-2 spike protein by immobilizing a multilayered nano-quencher of Nb_2_C MXene and carbon dot-modified aptamer probes of G-CDs@Apt onto a mixed cellulose ester (MCE) paper surface. The multilayered Nb_2_C MXene nano-quencher adsorbed abundant G-CDs@Apt probes and quenched their fluorescence signals based on an efficient FRET process, which could be recovered with the addition of SARS-CoV-2 spike protein targets. By utilizing the proposed Nb_2_C MXene/G-CDs@Apt paper sensor, highly sensitive and selective determination of SARS-CoV-2 spike protein was successfully achieved with a detection limit of 0.067 ng/mL (0.335 pg/test). The constructed paper-based sensor showed good anti-interference in both high- and low-salt detection conditions and had good practicability in detecting SARS-CoV-2 spike proteins in artificial saliva samples and real environmental samples.

However, there remain several challenges to be addressed: (i) The background fluorescence of the paper substrate under UV excitation has not been visually eliminated, which can limit the sensitivity of the visual detection. (ii) The supporting all-in-one microdevices for imaging and intelligent analysis of such paper-based sensors have not yet been developed. (iii) The paper-based MXene sensor awaits extension for the detection of other targets by replacing the aptamer. By exploring these directions, the proposed technology can be developed into a versatile and powerful tool in decentralized POC diagnosis, providing a new idea for the development of paper-based fluorescent sensors.

## Figures and Tables

**Table 1:** Recovery tests for SARS-CoV-2 spike protein detection in real river water samples (*n* = 3).

Samples	Added (ng/mL)	Determined (ng/mL)	Recovery (%)	RSD (%)
River water from Haihe River	0	Nd	/	/
	10	9.09 ± 0.31	90.87	3.41
	50	48.98 ± 0.73	95.96	1.52
	80	80.44 ± 1.63	100.55	2.02

Nd: not detected.

## Data Availability

Data will be made available upon request.

## Supplementary Materials

The following supporting information can be downloaded at: https://www.mdpi.com/article/10.3390/s25061637/s1, Figure S1: Characterization of the Nb_2_AlC precursor; Figure S2: Characterization of the Nb_2_C-MXene; Figure S3: Evaluation of stability of Nb_2_C-MXene/G-CDs@Apt at different storage time. Table S1: The main consumables used in the study; Table S2: EDX element analysis of Nb_2_AlC-MAX and Nb_2_C-MXene; Table S3: Raw cost accounting for the proposed Nb_2_C-MXene/G-CDs@Apt paper-based sensor; Table S4: Comparison of various analytical methods for spike protein detection.
