# Supplementary material for: Aptamer Paper-Based Fluorescent Sensor for Determination of SARS-CoV-2 Spike Protein
Source: Sensors (Basel). 2025 Mar 7;25(6):1637. doi: 10.3390/s25061637 (PMC11945200; doi:10.3390/s25061637)
Supplement: Supplementary file 1 [file sensors-25-01637-s001.zip › sensors-3424609-supplementary.pdf]

Aptamer Paper-Based Fluorescent Sensor for Determination of SARS-CoV-2 Spike Protein

Jincai Yang, Zunquan Zhao, Tianyi Ma and Jialei Bai *
Tianjin Key Laboratory of Risk Assessment and Control Technology for Environment and Food Safety, Military Medical Sciences Academy, Academy of Military Sciences, Tianjin 300050, China; jiasang27@163.com (J.Y.); zhaozunq2009@163.com (Z.Z.); matianyi2023@foxmail.com (T.M.)
* Correspondence: baijialeitj@163.com

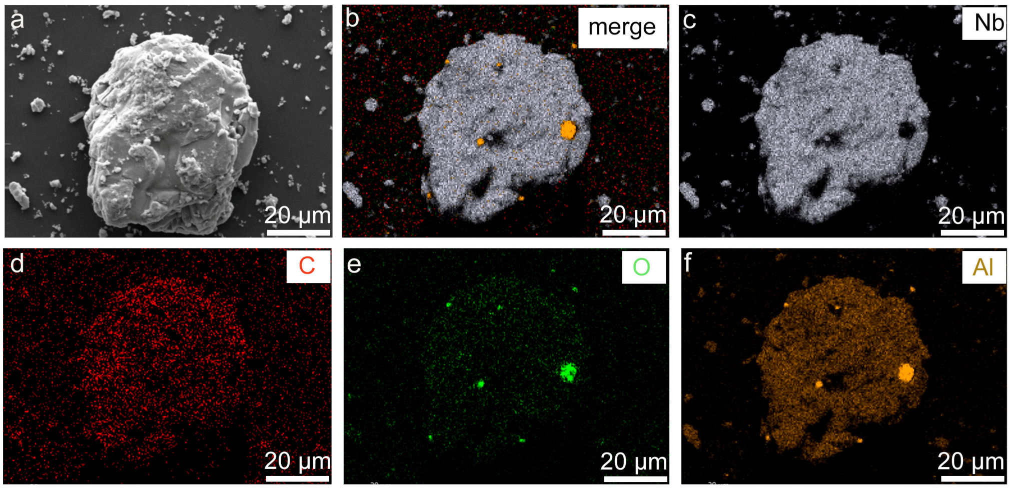

Figure S1. Characterization of the Nb_2AlC precursor. (a) SEM image of Nb_2AlC. (b–f) EDX mapping images of Nb_2AlC [Nb (Gray), C (red), O (green), and Al (brown)].

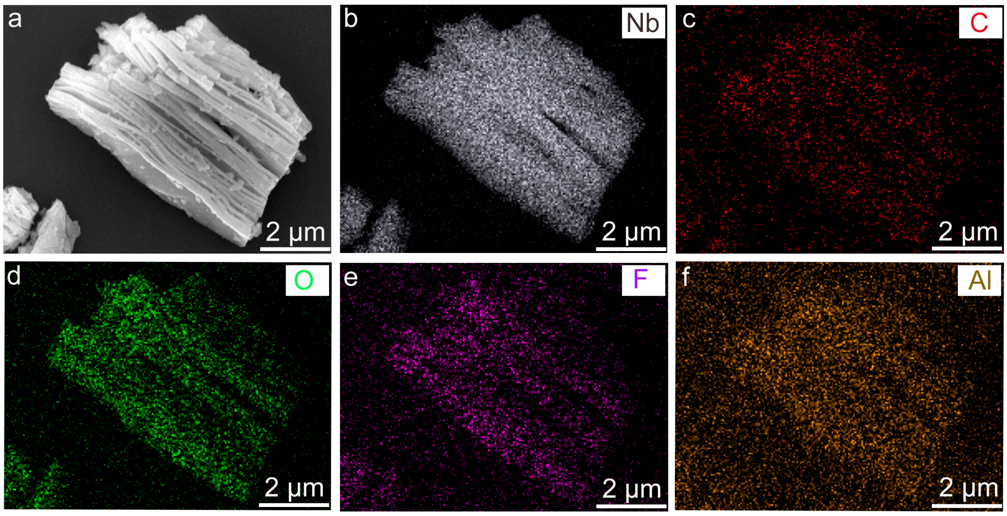

Figure S2. Characterization of the Nb_2C MXene. (a) SEM image of Nb_2C-MXene. (b–f) EDX mapping images of Nb_2C MXene [Nb (Gray), C (red), O (green), F (purple) and Al (brown)].

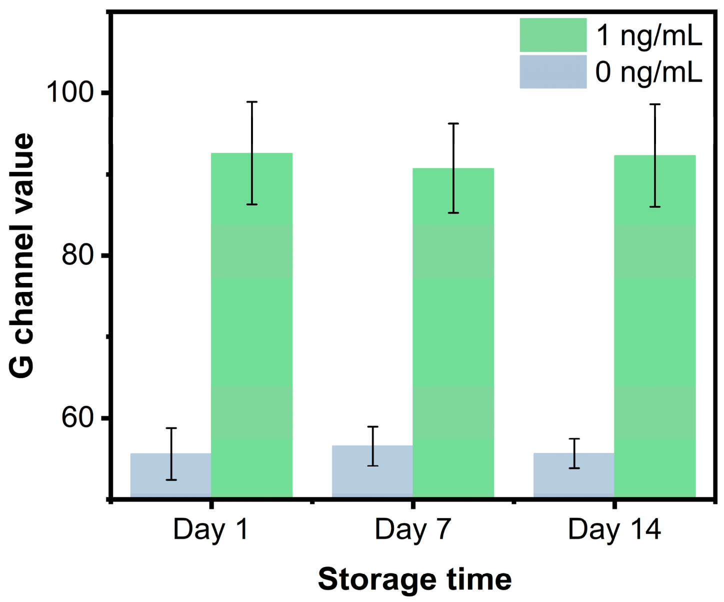

Figure S3. Evaluation of stability of Nb_2C-MXene/G-CDs@Apt at different storage time.

Table S1. The main consumables used in the study.

Consumables	Catalog	Parameter	Brand	Vendor
S1 aptamer	5204804939	/	Sangon Biotech	Shanghai Sangon Biotech Co.,Ltd.
S1 protein	D625026	100 μg	Sangon Biotech	Shanghai Sangon Biotech Co.,Ltd.
N protein	C500316-0100	100 μg	Sangon Biotech	Shanghai Sangon Biotech Co.,Ltd.
BSA	A602440	50 g	Sangon Biotech	Shanghai Sangon Biotech Co.,Ltd.
HB	A002402-0005	5 g	Sangon Biotech	Shanghai Sangon Biotech Co.,Ltd.
EDC	E106172	25 g	Aladdin Reagent	Tianjin Hejiaqi Bioreagents Co., Ltd.
NHS	H109330	25 g	Aladdin Reagent	Tianjin Hejiaqi Bioreagents Co., Ltd.
10X PBS	T494526	500 mL	Aladdin Reagent	Tianjin Hejiaqi Bioreagents Co., Ltd.
HF	H116236	500 mL	Aladdin Reagent	Tianjin Hejiaqi Bioreagents Co., Ltd.
Nb_2AlC	A980893	25 g	McLean Reagent	Tianjin Hejiaqi Bioreagents Co., Ltd.
G-CDs	/	5 mL	Mesolight	Tianjin Hejiaqi Bioreagents Co., Ltd.
Artificial saliva	R22154	500 mL	Yuanye Bio-Technology	Tianjin Hejiaqi Bioreagents Co., Ltd.
ELISA plate	F605034	96 well	Sangon Biotech	Tianjin Hejiaqi Bioreagents Co., Ltd.
Membrane Filter	MCE	0.22 μm	JINTENG	Tianjin Hejiaqi Bioreagents Co., Ltd.
Ultrafiltration tube	UFC5010	10 kDa	Milipore	Tianjin Hejiaqi Bioreagents Co., Ltd.

Table S2. EDX element analysis of Nb_2AlC-MAX and Nb_2C-MXene.

Element	Nb_2AlC-MAX		Nb_2C-MXene	
	Weight (%)	Atomic (%)	Weight (%)	Atomic (%)
C K	21.86	54.16	6.89	21.03
O K	7.7	14.31	16.41	37.59
Al K	11.46	12.64	7.00	9.51
Nb K	58.98	18.89	66.85	26.37
F K	/	/	2.85	5.50

Table S3. Raw cost accounting for the proposed Nb_2C MXene/G-CDs@Apt paper-based sensor.

Material	Price	Usage/test	Raw cost/test
S1 Apt	39.49 CHF/14 OD	2.5 pmol	0.017 CHF
G-CDs	61.98 CHF/5 mL	156.25 ng	0.00019 CHF
Nb_2C (Nb_2AlC)	CHF 61.91/1 g	937.5 ng	0.000058 CHF
MCE	22.31 CHF/100 pieces (diameter: 100 mm)	1 piece (diameter: 4 mm)	0.0044 CHF

Table S4. Comparison of various analytical methods for spike protein detection.

Analytical method	Raw Cost (CHF/test)	Linear range (ng/mL)	LOD (ng/mL)	Reference
Lateral flow immunoassay (LFIA)	9.42	/	166.66	[1]
Electrochemiluminescence immunosensor	9.53	10–10000	1.93	[2]
Electrochemical immunosensor	3.44	40–10000	19	[3]
Aptamer-based lateral flow devices	0.90	100–1000	51.81	[4]
MOF-Based Aptasensor	1.91	500–8000	72	[5]
Aptamer-based Electrochemical biosensor	0.77	0–5076	0.066	[6]
Aptasensor based on MXene and carbon dots	0.022	0.1–80	0.067	This work

References

1. Mohammad, S., Y. Wang, J. Cordero, C. Watson, R. Molestina, S. Rashid and R. Bradford. Development and validation of a rapid and easy-to-perform point-of-care lateral flow immunoassay (lfia) for the detection of sars-cov-2 spike protein. *Frontiers in Immunology* **2023**, 14, 1111644. https://doi.org/10.3389/fimmu.2023.1111644.
2. Hosseini, M., E. Sobhanie, F. Salehnia, G. Xu, H. Rabbani, M. Naghavi Sheikholeslami, A. Firoozbakhtian, N. Sadeghi, M. Hossein Farajollah, M. Reza Ganjali, et al. Development of sandwich electrochemiluminescence immunosensor for covid-19 diagnosis by sars-cov-2 spike protein detection based on au@bsa-luminol nanocomposites. *Bioelectrochemistry* **2022**, 147, 108161, https://doi.org/10.1016/j.bioelechem.2022.108161.
3. Fabiani, L., M. Saroglia, G. Galatà, R. De Santis, S. Fillo, V. Luca, G. Faggioni, N. D'Amore, E. Regalbuto, P. Salvatori, et al. Magnetic beads combined with carbon black-based screen-printed electrodes for covid-19: A reliable and miniaturized electrochemical immunosensor for sars-cov-2 detection in saliva. *Biosens. Bioelectron.* **2021**, 171, 112686. https://doi.org/10.1016/j.bios.2020.112686.
4. Wang, J., L. Zhao, X. Li, Y. Gao, W. Yong, Y. Jin and Y. Dong. Development of aptamer-based lateral flow devices for rapid detection of sars-cov-2 s protein and uncertainty assessment. *Talanta 2025*, 281, 126825. https://doi.org/10.1016/j.talanta.2024.126825.
5. Jiang, Z. W., T. T. Zhao, C. M. Li, Y. F. Li and C. Z. Huang. 2D mof-based photoelectrochemical aptasensor for sars-cov-2 spike glycoprotein detection. *ACS Applied Materials. Interfaces.* **2021**, 13, 49754-49761. https://doi.org/10.1021/acsami.1c17574.
6. Abrego-Martinez, J. C., M. Jafari, S. Chergui, C. Pavel, D. Che and M. Siaj. Aptamer-based electrochemical biosensor for rapid detection of sars-cov-2: Nanoscale electrode-aptamer-sars-cov-2 imaging by photo-induced force microscopy. *Biosens. Bioelectron.* **2022**, 195, 113595. https://doi.org/10.1016/j.bios.2021.113595.
